# Genetic and Gene-by-Environment Influences on Aggressiveness in Dogs: A Systematic Review from 2000 to 2024

**DOI:** 10.3390/ani15152267

**Published:** 2025-08-01

**Authors:** Stefano Sartore, Riccardo Moretti, Stefania Chessa, Paola Sacchi

**Affiliations:** Department of Veterinary Sciences, University of Turin, Grugliasco, 10093 Turin, Italy; stefano.sartore@unito.it (S.S.); stefania.chessa@unito.it (S.C.); paola.sacchi@unito.it (P.S.)

**Keywords:** dog behavior, aggressiveness, dog genetics, systematic review

## Abstract

Aggressiveness in dogs is a complex behavioral trait with implications for animal welfare and public safety. Despite domestication, dogs retain aggressive tendencies shaped by both genetic and environmental factors. This systematic review synthesizes the literature from 2000 to 2024 on the genetic and environmental bases of canine aggression. Using PRISMA 2020 guidelines, 144 articles were retrieved from Scopus and PubMed and screened in two phases, resulting in 33 studies selected for analysis. These were evaluated using a 20-question grid across seven categories, including phenotyping, genetic analysis, population structure, and future directions. The studies support a polygenic model of aggressiveness, with associations reported for genes involved in neurotransmission, hormone signaling, and brain function. However, inconsistencies in phenotyping, small sample sizes, and a limited consideration of environmental factors hinder robust conclusions. Most studies focused on popular companion breeds, while those commonly labeled as aggressive were underrepresented. The findings highlight the relevance of gene–environment interactions but underscore that aggression is often poorly defined and measured across studies. Future research should prioritize standardized phenotyping tools, broader breed inclusion, and the functional validation of genetic findings. These efforts will improve the understanding of dog aggression and inform breeding, behavioral assessment, and public policy.

## Simple Summary

This systematic review looks at why some dogs show aggressive behavior and whether it is influenced more by their genes or by their environment. Although dogs have lived alongside humans for thousands of years, they can still act aggressively, which can pose risks to people and other animals. The aim of this review was to understand what scientific research has discovered about the causes of aggression in dogs. The authors analyzed 19 studies published over the past two decades, focusing on how aggression was measured, what genetic differences were found, and what role environmental factors such as age, sex, or living conditions might play. They found that aggression is influenced by many genes and is not caused by just one factor. However, most studies had small sample sizes and relied on unclear ways to measure aggressive behavior, making it hard to draw firm conclusions. Some popular dog breeds were studied often, while those considered aggressive by the public were rarely included. The study concludes that more research is needed using consistent methods and a wider variety of breeds. Understanding the causes of aggression in dogs could lead to better training, breeding practices, and public safety measures, ultimately helping both people and animals.

## 1. Introduction

Aggressiveness can be considered a basic form of social interaction for wild animals, as it helps individuals access and defend resources that range from food to territory, mates to nesting sites, and all that concerns the survival of a given species. This attitude can show a broad spectrum of action, from simple signaling, in which animals can solve their rivalry by minimizing the physical risk, to direct aggression [1,2,3]. In the latter case, the consequences can be either severe injuries [4] or even death [5,6].

Domestication, irrespective of the species taken into account, is widely considered a two-way process between humans and animals, in which both partners obtain advantages from the relationship [7]. Dog domestication is an event arising from the wolf (*Canis lupus lupus*) and can be traced back over 15,000 years [8]. Therefore, according to our current knowledge, the dog is the first domesticated organism. Despite the fact that the level of intimacy between dogs and humans has increased in recent decades, there is archeological evidence testifying that this tight connection is quite ancient [9]. One of the expected evolutionary consequences of this process is the fact of dogs to ceasing their original behaviors in favor of more appropriate attitudes for coexistence with humans (e.g., tameness and docility [10]). One likely hypothesis of this behavior change is the neoteny theory, in which domestic animals behave like juvenilized versions of their wild counterpart, in the case of dogs, a juvenilized version of wolves [11]. Conversely, there is evidence that, despite a long period of domestication, the basic behavior of a species is not deleted but simply set aside, and thus it is ready to be expressed if or when necessary [12,13]. In view of this, aggressiveness is still present in dogs and, according to available data in the literature, it represents a serious public health issue in both the United States [14] and Europe [15,16]. The evidence that behavior variation comes from genes is provided by different surveys [17,18], but how a genetic variation specifically determines a particular behavior is only clear in regard to the so-called irreversible effects (e.g., the different abilities of the eyes, nose, or other organs affected by genes variation can lead to different abilities and cannot be reverted). On the other hand, the genetic mechanism intervening in the reversible effects, namely those behaviors affected by genes involved in pathways controlling hormones, hormone receptors, neurotransmitters, or receptors of neurotransmitters, and that can modify the behavior in front of different scenarios, is still under investigation [10,19]. After highlighting this difference within irreversible and reversible effects, we must contemplate that, to our current knowledge, aggressiveness is considered a reversible effect [20].

The aim of this work is to screen the scientific literature investigating gene variations and environmental factors involved in aggressive behavior in dogs and to subsequently organize the results in this systematic review. Furthermore, a secondary aim of this review would be to point out the ambiguity and inconsistency in phenotype definition when it comes to aggressivity and aggressive behaviors. Indeed, the idea behind this work is to try to clarify some interesting points toward a clearer interpretation of the available information in the literature on aggressiveness in dogs, which is (to the best of our knowledge) a scientific gap to be filled. To achieve this aim, we identified a total of 144 papers which was reduced to 33 after two screening steps. These same papers were than analyzed in this systematic review to address seven main topics, namely bibliographic information, general information, aggressivity phenotyping, genetic analysis, population genetics aspects, functional insights and evolutionary perspectives, study limitations, and future directions.

## 2. Materials and Methods

### 2.1. Literature Search and Exclusion Criteria

In order to compile this scientific literature review, a protocol for a literature search was set up according to the guidelines proposed by Preferred Reporting Items for Systematic reviews and Meta-Analyses (PRISMA) in its state-of-the-art statement version dated 2020 [21]. These guidelines are intended to help authors transparently report and publish systematic scientific reviews. The search was performed through Scopus, the scientific paper database from Elsevier (www.scopus.com, accessed on 3 June 2025), and PubMed, the scientific paper database from the American National Center for Biotechnology Information (NCBI, https://pubmed.ncbi.nlm.nih.gov/, accessed on 7 July 2025). Two different searches were performed at the beginning of June 2025 and during July 2025 on the same publishing date range (i.e., considering the publication years ranging from 2000 to 2024, with extremes included), considering scientific articles only, and using two different sets of keywords. The keywords were searched in the “article title, abstract, and keywords” field provided by Scopus and in the “all fields” query box in PubMed. The two keyword sets were as follows:

1. “dog* behaviour” AND “genetic*”;
2. “dog*” AND “aggressiv* behaviour” AND “genetic*”.

The *AND* Boolean operator in the search means that both terms included in the quotation marks must appear in the results, while the “*” operator is a so-called wildcard character which includes every other character that could eventually appear in the search string used. To better explain its usage, when using the “aggressiv*” notation in our search string the algorithm will include every possible word starting with the “*aggressiv*” root (e.g., aggressive, aggressiveness, aggressivity, and so on). The search produced a total of 143 records (Scopus: 34 and 47 papers for keyword sets 1 and 2, respectively; PubMed: 28 and 34 papers for keyword sets 1 and 2, respectively). The search reports were then downloaded in Excel format, consisting of a 2-way spreadsheet considering every paper as a record (i.e., as a row) and the selected descriptive information reported, each in a single column. The selected information consisted of the author names, titles, abstracts, publishing years, sources (i.e., scientific journals), and the language. A first round of data exclusion was performed based on missing data and/or papers written in languages other than English. To conclude this first exclusion step, duplicated papers in the dataset were removed (50 duplicates were found, originating from the four different queries).

**Figure 1:**
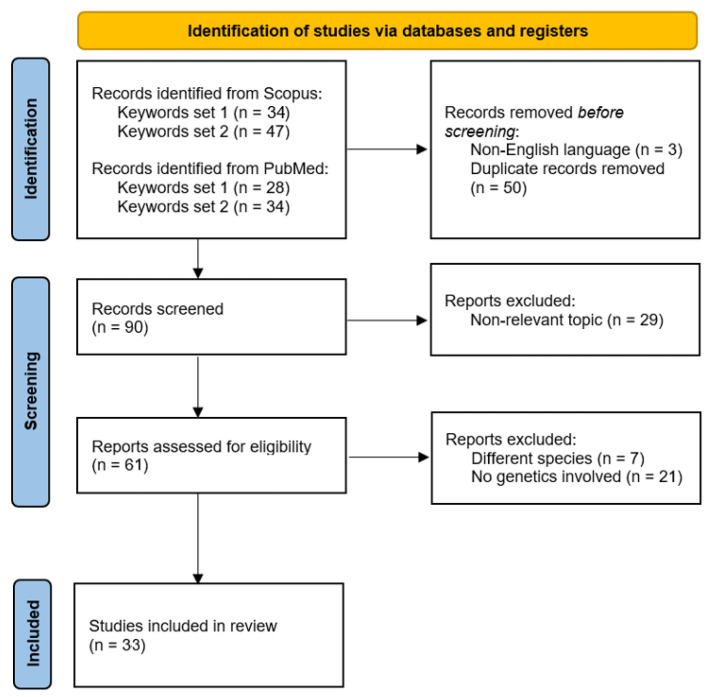
PRISMA 2020 flow diagram for new systematic reviews. In this diagram, the exclusion steps performed by the authors to select the corpus of papers to be analyzed to compile the present review are summarized.

A second exclusion round was manually performed by the authors by skimming the abstract of each paper, assessing the relevance of the articles to the investigated topic. In this round, 29 papers were discarded since they investigated non-relevant topics. Specifically, two confounding topics were detected and discarded: the former was abnormal and aggressive behavior in dogs due to rabies or other diseases, while the latter was “aggressiveness” which referred to the rapid formation and development of dogs’ tumors. Furthermore, 7 papers were removed because they involved other species (i.e., foxes, ferrets, and wolves). Of the remaining 61 papers addressing aggressive behavior, 21 were removed because they did not directly involve genetics. The PRISMA 2020 flow diagram for new systematic reviews (adapted from [21]), presenting the information reported above in a schematic fashion, is presented in Figure 1.

After these two exclusion rounds, a total of 33 topic-relevant scientific papers were selected and subsequently used to compile this review by the authors. A list of the scientific papers included in the review is reported in Table 1.

### 2.2. Analysis of Studies Included in Review

After the screening steps, a total of 33 scientific papers were selected and included in this review. These papers were read and analyzed by the authors using a list of 20 questions designed to extract the main information from each manuscript. The questions were divided into seven categories: (I) bibliographic information; (II) general information; (III) aggressivity phenotyping; (IV) genetic analysis; (V) population genetics aspects; (VI) functional insights and evolutionary perspectives; (VII) study limitations and future directions. The questions are reported in Table 2.

## 3. Results and Discussion

### 3.1. Question Analysis: (I) Bibliographic Information (Q1–2)

The majority of the papers (24 out of 33) had a European country as a first affiliation [13,22,23,24,25,26,27,28,29,30,32,33,34,35,36,37,39,40,41,43,44,46,48,49], with the remaining papers’ affiliations being divided into Asia [31,38,42,47,53] and North America [45,50,51,52], indicating that European countries are probably more aware regarding dogs’ aggressiveness, with 73% of publications compared to 27%. Within Europe, the highest number of papers published on the screened topic was from the Netherlands (6 out of 33, 18%), followed by Hungary (4 out of 33, 12%), and Norway and Sweden in joint third place (3 out of 33 each, 9%). Considering the Scandinavian Peninsula, 7 out of 33 (21%) papers in the analyzed corpus were from there. One possible explanation for this can be given by the greater attention that is paid in those latitudes to animal welfare and behavior. On the other hand, it is noteworthy that only two papers were from Mediterranean countries, namely one from Spain [23] and one from Italy [35]. Ultimately, the studies included in this review were published between 2005 and 2024, thus almost covering the entire analyzed publication date range (i.e., 2000–2024).

### 3.2. Question Analysis: (II) General Information (Q3–5)

Based on Q3 (i.e., “What is the main objective of the study?”), two main clusters have been identified in the 33 analyzed papers: (i) the biological basis (genetic and/or hormonal) of aggression and related behaviors (23 papers [22,23,26,28,29,30,31,32,33,34,36,39,40,42,43,44,45,49,50,51,52,53]); (ii) behavioral problem and breed-related studies (10 papers [13,24,25,27,35,37,38,41,46,47]). The first cluster includes studies aiming to identify the associations between specific genes, genetic markers, or genetic variants and aggressive behavior in dogs. It focuses on genetics, heritability, or neuroendocrine mechanisms underlying dogs’ behavior. Typically, the goal of the papers in this cluster is to understand how genetic variation in candidate genes contributes to aggressive behaviors. This first cluster involves papers published through the whole publication range (i.e., the cluster publication range of 2005–2024). The second cluster focuses on the investigation of behavioral problems (mainly aggression and fear-related behaviors) and the genetic architecture of canine behavioral traits across breeds or populations. This cluster includes papers published across most of the included publication range, with papers spanning from 2006 to 2021. Moving to Q4 (i.e., “What type of study was conducted?”), three main approaches have been employed in the scientific literature so far: (i) genomic or molecular studies (24 papers [22,23,26,28,29,30,31,32,33,34,36,37,39,40,42,43,44,45,46,48,49,50,51,52]); (ii) experimental/comparative behavioral testing (4 papers [24,43,47,53]); (iii) questionnaire-based behavioral surveys (12 papers [13,25,27,31,35,38,41,43,44,45,50,51]). In this case, some papers were clustered in more than one group (e.g., [43]). The first cluster incorporates genetic, genomic, or endocrine data, features behavior as a phenotype, and aims at the study of causal links between molecular variation and behavior. In contrast, in the second cluster, the different studies involved standardized behavioral tests to assess behavior under replicable conditions. Lastly, the third cluster focuses on owner-reported behaviors collected through questionnaires, which represent a low-cost, high-sample-size approach to address canine behavior. The three clusters are well represented throughout the whole considered publication range, with questionnaire-based studies appearing from 2010 onward.

Lastly, Q5 (i.e., “What is the sample size and which breeds were analyzed?”) investigated the number of animals involved in the papers and the mentioned dog breeds. The analysis showed a wide range in sample sizes, from small single-breed studies to large-scale population analyses involving thousands of individuals. Twelve studies included single breeds, while 14 focused on multiple breeds or mixed-breed dogs. A total of 16 dog breeds were specifically mentioned, although many papers involved datasets with a high number of breeds. Among the directly mentioned breeds, Golden Retrievers and Labrador Retrievers were the most popular ones (mentioned in six papers each), followed by German Shepherds (five papers). Early studies (2005–2010) were mostly focused on single-breed studies, while a shift toward multi-breed and larger-sample studies was observed after 2010. Most recent studies (2020–2024) involved mainly multi-breed and comparative approaches.

### 3.3. Question Analysis: (III) Aggressivity Phenotyping (Q6–7)

The following questions were used to analyze the key point of how the aggressivity phenotype was defined. Through Q6 (i.e., “How is aggressivity defined in the study?”), different approaches and definitions of aggressivity were identified. From this analysis, it is clearly shown that there is a high variability in what is considered aggressivity or aggressive behavior. Indeed, many studies defined aggression behaviorally (using terms like growling, snapping, biting), while others used a subtype-specific definition based on the target of the aggressive behavior (e.g., stranger-, owner-, or dog-directed aggressivity). Two main clusters were defined in papers directly defining aggressivity: (i) operational or trained/professionist-assessed definitions (13 papers [23,24,29,30,34,36,37,42,43,47,48,52,53]); and (ii) general or owner-reported aggression (20 papers [13,22,25,26,27,28,31,32,33,35,38,39,40,41,44,45,46,49,50,51]). The difference between the two clusters lies in the scientific soundness of the definition of aggressivity presented in the paper: in the former cluster can be found studies providing a clear, structured, and standardized definitions, while in the latter, the definition is either generally or subjectively described. Question Q7 (i.e., “What methods were used to assess aggression?”) analysis showed different approaches in how to assess aggressivity, ranging from behavioral questionnaires, to direct experimental observation in a controlled environment, to indirect or no formal measurement, consistent with the answers from Question Q6. When aggressivity was directly assessed, two main clusters were defined: (i) questionnaire-based assessment, with a large use of CBARQ surveys developed by [54] (17 papers [13,25,26,27,28,31,32,33,35,38,39,41,42,45,46,50,51]); (ii) experimental or observational structured testing performed by researchers or trained observers (13 papers [23,24,29,30,34,36,37,40,42,47,48,52,53]); and (iii) other (3 papers [22,44,49]). As described before, a rise in questionnaire-based studies was observed after 2010, with the introduction of structured surveys.

### 3.4. Question Analysis: (IV) Genetic Analysis (Q8–13)

The following group of questions revolve around the genetic analysis performed in the scientific papers reviewed. Specifically, the techniques used were analyzed in Q8 (i.e., “What genetic markers or techniques were used?”). Five main clusters were identified by the similar contents of the answers: (i) no genetic analysis performed (10 papers [13,23,24,25,27,35,38,41,47,53]); (ii) pedigree-based genetic models with no molecular data (3 papers [26,29,37]); (iii) candidate gene studies (9 papers [31,33,34,36,40,42,43,44,48]); (iv) genome-wide and high-throughput analyses (8 papers [22,28,39,45,46,49,50,51]); (v) functional or validation studies (3 papers [30,32,51]). Moving to answer Q9 (i.e., “Were any specific genes or genomic regions associated with aggression identified?”), significant associations were found in 18 out of 33 papers; that is slightly more than half of the screened papers [30,31,32,33,34,36,39,40,42,43,44,45,46,48,49,50,51,52]. The identified genetic variations were different among the papers, as different candidate genes or regions were considered by the analyzed studies, thus leading to the answer for Q10 (i.e., “Did the study find evidence for polygenic inheritance, or was a single major gene involved?”): the complexity of the aggressivity phenotype is most likely due to a polygenic inheritance mechanism, in which many different genes and genetic regions could be involved in the observed differences. Indeed, 25 out of 33 papers (76%) agreed on suggesting a polygenic underlying mechanism [23,25,26,27,28,29,30,31,32,33,34,36,37,38,39,40,42,43,44,45,46,49,50,51,52], while the remaining 7 did not directly address the issue [13,22,24,35,41,47,53]. Lastly, only one paper [48] supported a single major gene position. The next three questions were related to the models used in the studies, specifically referring to the direct comparison between aggressive and non-aggressive individuals (i.e., Q11), and the environmental factors considered and their possible interactions with genetics (i.e., Q12 “Were any environmental factors considered?”, and Q13 “Were gene–environment interactions considered?”, respectively). The majority of the papers performed a direct comparison between aggressive and non-aggressive subjects (28 out of 33 papers, 85%) [13,25,26,27,28,30,31,32,33,34,35,36,37,39,40,41,42,43,44,45,46,47,48,49,50,51,52,53]. Regarding environmental factors, the most commonly accounted environmental factors were age, sex, neuter status/reproductive status, and housing, along with other minor factors seldomly considered (e.g., litter size/composition, role as pet vs. working dog, or owner-related factors). While only 6 out of 33 papers (18%) did not account for any of them [22,27,34,46,48,53], the majority of the papers either directly incorporated environmental factors in a statistical model or used them as screening or exclusion criteria. Lastly, only three papers accounted for gene-by-environment interactions [37,43,44].

### 3.5. Question Analysis: (V) Population Genetics Aspects (Q14–15)

From a population genetics point of view, only 8 out of 33 papers (24%) estimated the heritability of the aggressivity phenotype studied (Q14, “Was heritability estimated?”) [23,26,29,37,39,45,50,52]. The heritability values reported are extremely variable, as they were estimated from different measurement approaches and on variable phenotype definitions. Regarding the second question of this group (Q15, “Were differences between breeds or within-breed variation examined?”), only 14 out of 33 papers (42%) directly discussed the observed differences between breeds [13,22,24,29,30,36,37,38,40,44,45,49,50,53].

### 3.6. Question Analysis: (VI) Functional Insights and Evolutionary Perspectives (Q16–18)

A few papers further expanded their investigation by adding functional studies in order to validate genetic findings, namely 6 out of 33 (18%) [24,30,33,45,49,51], as investigated in Q16 (“Were any functional studies performed to validate genetic findings?”). In contrast, a higher proportion tried to discuss them in an evolutionary light (23 out of 33, 70%) as assessed via Q17 (“Did the study discuss evolutionary or selective pressures on aggression-related genes?”) [22,24,25,29,30,31,34,35,36,37,38,40,42,43,44,45,46,48,49,50,51,52,53]. The complexity of functional studies in terms of experimental design and realization, along with matters of time and funding requirements, is the main factor that is likely responsible for this deficiency.

Lastly, our attention focused on those studies that pursued a cross-species comparison approach (Q18, “Were cross-species comparisons made?”), since many aggressivity-related issues can be found in various species, humans included. Only 10 out of 33 papers (30%) did not reference any other species, with a dog-specific design of the study and interpretation of the results [13,23,26,29,35,37,41,47,50,53]. In contrast, most of the studies indeed used cross-species comparisons (e.g., with humans, mice, foxes) to support their hypothesis or interpretation of results.

### 3.7. Question Analysis: (VII) Study Limitations and Future Directions (Q19–20)

The last category referred to the limitations and the future perspectives mentioned in the papers analyzed. The former information has a pivotal role in attesting the limitations and weaknesses of the screened papers, while the latter offers an holistic view on the directions in which this field of study is going. Regarding the main limitations (Q19, “What were the main limitations of the study?”), the most common ones were a small sample size and limited breed diversity [13,23,24,26,28,30,31,32,33,34,36,39,40,42,43,44,46,47,48,51,52,53]. These two limitations often result in a constraint on statistical power and reduced generalizability. A third important limitation was the reliance on owner-reported behavior or subjective scoring systems [13,23,24,25,27,31,35,38,39,41,42,50,51]. This limitation strongly affects phenotyping and behavioral classification as biases could be created due to a possible owner-distorted point of view. Furthermore, it reflects the challenge of accurately capturing behavioral traits. Future research directions, as suggested by the authors in their own studies (Q20, “Did the authors propose any future research directions or practical applications?”), point toward recurrent themes. Most studies affirm that behavior, particularly aggression and social traits, has a heritable component, often polygenic in nature. These studies, therefore, could be useful as a foundation for future genetic studies and could help in the process of discovering the multifactorial basis (most likely influenced by both genetic and environmental factors) of aggressive behavior. In addition, the importance of future findings in this topic is also related to the use of dogs as a model organism for human social behavior. A third important consideration that emerged through this analysis is the impact of domestication on dogs’ behavior and the possible application of the same selection towards future behavior-informed breeding, leading to improved welfare for both pets and owners. Lastly, multiple papers confirm the validity of structured behavioral tools (e.g., the CBARQ and similar surveys) for reliable phenotyping. All these future directions and observed limitations highlight the need to understand how genes affect behavior from a biological point of view, as well as putting strong emphasis on expanding sample sizes/numbers of breeds. Furthermore, a focus on real-world implications like breeding practices, behavioral screening, or comparative medical relevance is highly desirable.

## 4. Conclusions

This review is focused on genetic bases of dog aggressivity and according to the 33 selected articles used for its drafting, we can draw the following main conclusions: irrespective of the fact that dogs have been domesticated numerous years ago, they still maintain aggressivity as part of their temperament. This behavior can become difficult to handle when it shifts into hyper-aggressivity that can be directed to both humans and non-humans. Aggressivity is a complex trait in which different genes, environmental factors (e.g., maternal effect, sex, age, neuter status, litter size and owner personality), and their interactions play a pivotal role; in this regard, some significant associations were detected by some authors; conversely, the heritability of the trait has been estimated in few surveys so far. The main limitations of all the investigations taken into account for this review are mostly sampling, testing reliability, and phenotyping and behavioral classification issues. In the latter concern, the Canine Behavioral Assessment and Research Questionnaire (CBARQ) was the most employed tool but, in general, irrespective of the method used (owner interviews, veterinarians interviews, standardized tests), phenotyping and behavioral assessment is still the main weakness of aggressiveness classification. Therefore, the need for improving this issue appears to be crucial from the perspective of a better and clearer classification of aggressive phenotyping.

## Figures and Tables

**Table 1:** List of the 33 selected papers for this review analysis.

First Author	Publication Date	Journal	Doi	Reference Number
L van den Berg	2005	Journal of Heredity	10.1093/jhered/esi108	[22]
J Pérez-Guisado	2006	Applied Animal Behaviour Science	10.1016/j.applanim.2005.11.005	[23]
K Svartberg	2006	Applied Animal Behaviour Science	10.1016/j.applanim.2005.06.014	[24]
L van den Berg	2006	Behavior Genetics	10.1007/s10519-006-9089-0	[25]
A-E Liinamo	2007	Applied Animal Behaviour Science	10.1016/j.applanim.2006.04.025	[26]
J Våge	2008	Journal of Veterinary Behavior	10.1016/j.jveb.2008.05.003	[27]
L van den Berg	2008	Behavior Genetics	10.1007/s10519-007-9179-7	[28]
EH van der Waaij	2008	Journal of Animal Science	10.2527/jas.2007-0616	[29]
K Hejjas	2009	Genes, Brain and Behavior	10.1111/j.1601-183X.2008.00475.x	[30]
Y Takeuchi	2009	Animal Genetics	10.1111/j.1365-2052.2009.01888.x	[31]
J Våge	2010a	BMC Veterinary Research	10.1186/1746-6148-6-34	[32]
J Våge	2010b	Genes, Brain and Behavior	10.1111/j.1601-183X.2010.00568.x	[33]
WS Proskura	2013	Acta Veterinaria Brno	10.2754/avb201382040441	[34]
S Diverio	2014	Journal of Veterinary Behavior	10.1016/j.jveb.2014.02.007	[35]
A Kis	2014	PLOS One	10.1371/journal.pone.0083993	[36]
A-S Sundman	2016	Genes, Brain and Behavior	10.1111/gbb.12317	[37]
A Tonoike	2016	Scientific Reports	10.1038/srep17710	[38]
J Ilska	2017	Genetics	10.1534/genetics.116.192674	[39]
E Kubinyi	2017	Frontiers in Psychology	10.3389/fpsyg.2017.01520	[40]
JAM van der Borg	2017	Applied Animal Behaviour Science	10.1016/j.applanim.2017.06.004	[41]
A Konno	2018	Journal of Heredity	10.1093/jhered/esy012	[42]
K Kovács	2018	Frontiers in Psychology	10.3389/fpsyg.2018.00435	[43]
ME Persson	2018	PeerJ	10.7717/peerj.5889	[44]
EL MacLean	2019	Proceedings of the Royal Society B	10.1098/rspb.2019.0716	[45]
J Friedrich	2020	Advanced Genetics	10.1002/ggn2.10024	[46]
YK Kim	2021	Journal of Veterinary Behavior	10.1016/j.jveb.2020.10.001	[47]
S Mikkola	2021	Scientific Reports	10.1038/s41598-021-88793-5	[13]
D Polasik	2021	Acta Veterinaria Brno	10.2754/avb202190030295	[48]
S Shan	2021	Frontiers in Veterinary Science	10.3389/fvets.2021.693290	[49]
K Morrill	2022	Science	10.1126/science.abk0639	[50]
AR Sanders	2022	Frontiers in Psychology	10.3389/fpsyg.2022.1025494	[51]
N Bhowmik	2024	Genes	10.3390/genes15121611	[52]
M Nagasawa	2024	Peptides	10.1016/j.peptides.2024.171224	[53]

**Table 2:** List of questions used to analyze the selected papers.

Number	Category	Question
1	Bibliographic information	First author’s affiliation?
2		Year of publication?
3	General information	What is the main objective of the study?
4		What type of study was conducted?
5		What is the sample size and which breeds were analyzed?
6	Aggressivity phenotyping	How is aggressivity defined in the study?
7		What methods were used to assess aggression?
8	Genetic analysis	What genetic markers or techniques were used?
9		Were any specific genes or genomic regions associated with aggression identified?
10		Did the study find evidence for polygenic inheritance, or was a single major genes involved?
11		Were comparisons made between aggressive and non-aggressive individuals?
12		Were any environmental factors considered?
13		Were gene–environment interactions considered?
14	Population genetics aspects	Was heritability estimated?
15		Were differences between breeds or within-breeds variation examined?
16	Functional insights and evolutionary perspectives	Were any functional studies performed to validate genetic findings?
17		Did the study discuss evolutionary or selective pressures on aggression-related genes?
18		Were cross-species comparisons made?
19	Study limitation and future directions	What were the main limitations of the study?
20		Did the authors propose any future research directions or practical applications?

## Data Availability

Data are contained within the article.
